# Response to “Acute Hepatitis Induced by Lyprinol, the Lipid Extract of the Green-Lipped Mussel (*Perna canaliculus*), in a Patient with Polyarthrosis”

**DOI:** 10.1155/2014/692043

**Published:** 2014-08-20

**Authors:** Stephen P. Myers, Christopher J. Oliver

**Affiliations:** ^1^NatMed-Research, Southern Cross University, Australia; ^2^Blackmores Institute, Australia

The title of the case report “Acute hepatitis induced by lyprinol, the lipid extract of the green-lipped mussel (*Perna canaliculus*), in a patient with polyarthrosis” [1] asserts a definitive causal relationship between Lyprinol and acute hepatitis. We are concerned that the report itself does not provide evidence that supports a definitive or highly probable association. As no rechallenge was undertaken definitive causality is impossible to prove.

The Council for International Organisations of Medical Sciences and Roussel Uclaf Causality Assessment Method (CIOMS/RUCAM) scale for drug-induced hepatotoxicity [2, 3] gives a score between 2 and 5 (see Table 1). The difference between these scores is dependent on information missing from the case report. At best this case report demonstrates that a causal association is possible. The Naranjo scale which is used to determine the likelihood of an adverse drug reaction [4] confirms this finding, giving a score of +2 which indicates the reaction is possible, as opposed to probable or definitive.

The subject had a six month history of recurrent, right sided epigastric pain radiating to her right shoulder and back and presented with an acute acerbation of this abdominal pain. On ultrasound of the gall bladder several concrements were found and it is therefore not possible to rule out that the episode of acute abdominal pain could be an episode of choledochal lithiasis.

The strength of the association in a single case report is determined by the absence of potential confounding factors. An important potential confounder in this case is that pantoprazole, which was being taken concomitantly with Lyprinol, has itself been the subject of multiple case reports of acute liver toxicity [5–7]. This confounder appears to be ruled out by the authors as the medication was stable at the time of the acute hepatitis. It is plausible, however, that an acute inflammatory flare occurred in an individual with known polyarthropathy and that this primed the liver toxicity in what had otherwise been a well-tolerated medicine. It is also possible that if pantoprazole was withdrawn at the same time as Lyprinol (not stated in case report) this may have caused the observed reduction in liver enzymes and improvement in the clinical picture, not the withdrawal of Lyprinol as was suggested.

Absent from the case report was basic information such as dosage of Lyprinol taken by the subject; indication of her height and weight; the specific diagnosis of her polyarthropathy; its contemporary history; and biological measures of its current severity (ESR and/or CRP).

Lyprinol is an extract of the green-lipped mussel which was a traditional staple of the diet of the New Zealand Maori people for hundreds of years and remains commonly eaten throughout New Zealand and Australia. It has been demonstrated to have anti-inflammatory activity [8] and is not an obvious biological candidate for inducing direct liver toxicity. To date outside of the report by Abdulazim et al. only two other cases of hepatoxicity have been described in the literature and both of these cases had a weak association [9]. PharmaLink, the manufactures of Lyprinol, report that they have sold 700 million Lyprinol capsules in 39 countries over the past twelve and half years (McLean, N. PharmaLink Director. Personal Communication, August 2013). The low biological plausibility for direct toxicity and the small number of cases in a widely used dietary supplement suggests that any real hepatotoxic reaction has a high likelihood of being idiosyncratic.

A definitive causal relationship was not demonstrated in this case report and the paper's title is misleading.

## Figures and Tables

**Table 1:** CIOMS/RUCAM score for the case report on acute hepatitis induced by Lyprinol.

Scale item	Scoring criteria	Score
(1) Time of onset	Time from drug intake to reaction: first exposure 5–90 days	+2
(2) Course	Greater than 50% improvement in 8 days	+3
(3) Risk factors	Age over 55 years +1/alcohol—unknown	+1
(4) Concomitant drugs	Concomitant drug known as hepatotoxin and with comparable or suggestive time of onset	−2
(5) Alternative nondrug causes∗	Group 1: HAV serology +ve/alcoholism—unknown = 0 Group 2: Auto-immune polyarthropathy possible = −3	−3 to 0
(6) Previous hepatotoxic information	Reaction published but unlabelled	+1
(7) Response to rechallenge	Not undertaken	0

Total score		2–5

Interpretation: unlikely (2) to possible (5)		

∗ *Explanation for variation on scale item 5—alternative nondrug causes*: the RUCAM outlines two groups of nondrug causes of acute hepatitis that need to be considered which are divided into two groups. Group 1 consists of recent HAV, HBV, or HCV; biliary obstruction; alcoholism; or history of hypotension (particularly if there is underlying heart disease). Group 2 consists of complications of underlying disease and clinical or biological context for CMV, EBV, or herpes virus infection. A score of 0 is given for 5 or 4 of Group 1 being excluded and −3 given if a nondrug cause such as acute autoimmune polyarthropathy is highly probable.
